# Andrographolide Inhibits Cholangiocarcinoma Cell Migration by Down-Regulation of Claudin-1 *via* the p-38 Signaling Pathway

**DOI:** 10.3389/fphar.2019.00827

**Published:** 2019-07-25

**Authors:** Phorutai Pearngam, Supeecha Kumkate, Seiji Okada, Tavan Janvilisri

**Affiliations:** ^1^Department of Biochemistry, Faculty of Science, Mahidol University, Bangkok, Thailand; ^2^Department of Biology, Faculty of Science, Mahidol University, Bangkok, Thailand; ^3^Division of Hematopoiesis, Joint Research Center for Human Retrovirus Infection & Graduate School of Medical Sciences, Kumamoto University, Kumamoto, Japan

**Keywords:** cholangiocarcinoma, andrographolide, migration, claudin, natural product, metastasis

## Abstract

Andrographolide, a bioactive phytochemical from *Andrographis paniculata*, is emerging as a promising anticancer agent against various cancers. This study aims to investigate anticancer activities of andrographolide against cholangiocarcinoma (CCA) and to understand the underlying mechanism. The anti-proliferative activity of andrographolide was evaluated in a range of cholangiocarcinoma (CCA) cell lines including HuCCA-1, KKU-100, KKU-M213, and RMCCA-1. The anti-migration activity and the corresponding mechanism were studied in highly metastatic KKU-M213 cells. The results indicated that andrographolide significantly inhibited the proliferation of CCA cells with the 50% inhibitory growth concentration (IC_50_) of ∼120 µM. Andrographolide also inhibited CCA cell migration and invasion. Our further explorations demonstrated that andrographolide decreased the expression of claudin-1, a major tight junction protein, while it up-regulated the expression of Snail, a transcriptional repressor of claudin-1. Moreover, andrographolide induced the phosphorylation of Jun N-terminus kinase (JNK) and p-38 Mitogen-activated protein kinase (MAPK). Treatment with the p-38-specific inhibitor recovered the claudin-1 expression and migration ability of CCA cells. This work demonstrated the potential anticancer effects of andrographolide, indicating that andrographolide could inhibit CCA cell migration *via* suppression of claudin-1 through the activation of p-38 MAPK signaling pathway. This compound would be useful for development of alternative therapeutic agent for CCA.

## Introduction

Cholangiocarcinoma (CCA) is a cancer that arises in the epithelium of biliary tree, both intra-hepatic and extra-hepatic bile ducts (Gores, 2003). The worldwide incidence of this cancer is relatively rare than are other gastrointestinal cancers (Shaib and El-Serag, 2004). However, high prevalence has been recorded in the Southeast Asian region, in particular the north-eastern part of Thailand (Sripa and Pairojkul, 2008). The mortality rate of CCA patients is high due to late clinical presentation. The primary treatment strategy for CCA is surgical resection of the tumor. However, only some cases are applicable for surgery, posing a limitation of the therapeutic options. The 5-year survival rate after resection is very low (20–30%) (Rosen et al., 2010). Most conventional chemotherapeutic agents such as gemcitabine, cisplatin, paclitaxel, and oxaliplatin have very limited efficiency in CCA patients and also could induce chemo-resistance (Lee et al., 2008; Valle et al., 2010; Weigt and Malfertheiner, 2010; Yang et al., 2013). Thus, it is urgently needed to find alternative treatments for CCA.

Poor clinical outcomes for CCA are largely due to metastatic disease. Metastatic processes in tumor cells involve a series of independent steps including cell protrusion, adhesion formation, detachment, and cell contraction (Lauffenburger and Horwitz, 1996; Friedl and Brocker, 2000). These steps are regulated by complicated molecular mechanisms. Intracellular signaling pathways including the p-38 MAPK and Jun N-terminus kinase (JNK) are ubiquitously activated in most cancer types and play a vital role in cancer progression and metastasis. However, the understanding of p-38 MAPK and JNK in cancer whether they are either tumor suppressor or oncoproteins is still unclear (Wagner and Nebreda, 2009). Certain proteins involved in epithelial–mesenchymal transition (EMT) have also been widely reported to be responsible for cancer metastasis (Thiery, 2002). The typical events occurring in cell migration include a loss of adherent E-cadherin and an increase in vimentin expression (Lamouille et al., 2014). Moreover, claudin-1, a major tight junction protein, has been associated with metastasis. The high expression of claudin-1 has been found in the aggressive cancer tissue, and the knockdown of claudin-1 reduced cell migration (Blanchard et al., 2013). Repression of claudin-1 has been strongly correlated with the enhanced expression of negative transcription factors for EMT proteins (Peinado et al., 2007).

In recent years, active compounds from natural products have gained attention for potential use in interventions against cancer progression and metastasis. Andrographolide, a diterpenoid lactone derived from *Andrographis paniculata* Nees, a herbal medicine widely used across Southeast Asian countries (Sareer et al., 2014) has been shown to exhibit a broad range of pharmacological properties (Gupta et al., 2017; Islam, 2017; Yang et al., 2017), and many recent studies have revealed its promising anticancer effects (Lim et al., 2012; Mishra et al., 2015). It has been shown to suppress cancer cell proliferation by disrupting proteins related to cell cycle regulation and to inhibit MCF-7 breast cancer cell proliferation by inducing G1 cell cycle arrest through increased p27 and decreased CDK4 (Rajagopal et al., 2003). In addition, this compound has been shown to promote apoptosis (Cheung et al., 2005). It has been demonstrated that andrographolide also inhibits cancer cell migration and invasion by interfering expression of proteins or cellular signaling pathways that play a key role in cancer metastasis. Its migratory inhibition on human non-small cell lung cancer A459 cells was mediated through down-regulation of PI3K/Akt signaling pathway that contributes to reduced expression of MMP-7, an extracellular matrix degradation enzyme (Lee et al., 2010). For CCA, crude ethanolic extract from first true leaf stage *A. paniculata* has previously been reported to inhibit cell proliferation and to induce apoptosis in HuCCA-1 and RMCCA-1 cells (Suriyo et al., 2014). However, the effects of purified form of andrographolide on other relevant cancer properties including migration and invasion remain elusive. In this study, we therefore examined the anticancer activities of andrographolide in a range of CCA cells focusing on migration and invasion ability of CCA cells and elucidated the underlying molecular mechanisms.

## Materials and Methods

### Chemicals and Antibodies

Andrographolide (98% purity), ribonuclease A, and phenylmethylsulfonyl fluoride (PMSF) protease inhibitor were purchased from Sigma Chemicals (Sigma-Aldrich, St. Louis, MO, USA). 3-(4,5-Dimethylthiazol-2-yl)-2,5-diphenyltetrazolium bromide (MTT) was purchased from United States Biological (Massachusetts, MA, USA). Propidium iodide (PI), Ham’s F-12 nutrient, and 10,000 U/ml penicillin/streptomycin were obtained from Invitrogen (Life Technologies, Grand Island, NY, USA). Fetal bovine serum (FBS) was purchased from Thermo Scientific Hyclone (South Logan, UT). BD Matrigel^™^ matrix growth factor reduced (GFR) was purchased from BD Bioscience (San Jose, CA, USA). The TMB sure blue substrate was obtained from KPL (Gaithersburg, MD, USA). Bradford solution was purchased from Bio-Rad (Hercules, CA, USA). Antibodies against actin, Akt, phospho-Akt (Ser473), Erk1/2, phospho-Erk1/2, p-38, phospho-p-38, JNK, phospho-JNK, and antibodies against EMT proteins as well as horseradish peroxidase (HRP)-conjugated anti-rabbit and anti-mouse secondary antibodies were from Cell Signaling Technologies (Beverly, MA, USA). For immunofluorescence, claudin-1 antibody was purchased from Santa Cruz Biotechnology (Santa Cruz, CA, USA), and anti-mouse antibody conjugated with Alexa Fluor 594 was from Thermo Fisher Scientific (Waltham, MA, USA). The p-38 MAPK SB 203580 inhibitor and JNK inhibitor SP600125 were purchased from Abcam Chemicals (Cambridge, UK).

### Andrographolide Stock Solution Preparation

Andrographolide was dissolved in DMSO at a concentration of 100 mM as a stock solution and stored at −20°C. Andrographolide solution at the desired concentrations was freshly prepared by diluting from a stock solution in serum-free Ham’s F-12 media. Control experiments received only media and the same amount of DMSO. The final concentration of DMSO was adjusted to 1% for all andrographolide concentrations.

### Cell Culture

The CCA cell lines, HuCCA-1 (Sirisinha et al., 1991) and RMCCA-1 (Rattanasinganchan et al., 2006), were kindly gifted from Prof. Stitaya Sirisinha and Assoc. Prof. Rutaiwan Tohtong, Faculty of Science, Mahidol University, respectively. KKU-100 (Sripa et al., 2005) and KKU-M213 (Uthaisar et al., 2016) CCA cell lines were purchased from the Japanese Collection of Research Bioresources Cell Bank. These cells were cultured in Ham’s F-12 medium supplement with 10% FBS and 100 U/ml of penicillin/streptomycin. All cell lines were maintained in a moisture incubator at 37°C with 5% CO_2_.

### Cell Viability by MTT Assay

Cells were seeded at a density of 2 × 10^4^ cells in 100 µl of medium and were exposed to different concentrations of andrographolide ranging from 0 to 200 µM for 48 h. After incubation, 10 µl of MTT solution (5 mg/ml) was added, and the cells were further incubated for 3 h. The production of formazan was solubilized by adding 100 µl of DMSO. The absorbance was measured by spectrophotometry at 570 nm (JASCO model FP-6200, MD, USA). The percentage of cell viability was calculated relative to the untreated control cells.

### Cell Cycle Analysis

CCA cells were seeded into 24-well plates at a density of 1.5 × 10^5^ cells per well in 1 ml of medium and then treated with 0, 25, 50, and 100 µM of andrographolide for 48 h. Cells were washed with PBS and trypsinized prior to fixation with chilled 70% ethanol for 1 h. Following the incubation of ribonuclease A (100 µg/ml) at 37°C for 30 min, cells were stained with 25 µg/ml of propidium iodide and further incubated for 30 min at 37°C in the dark. Cell cycle was analyzed using FACSCanto flow cytometer (BD Bioscience) with FACSDiva program from a minimum of 30,000 cells.

### Wound Healing Assay

CCA cells were seeded onto 24-well plates at a density of 1.5 × 10^5^ cells in 1 ml of medium and cultured at 37°C with 5% CO_2_ for 48 h. Following the confluence, medium was removed, and the cells were treated with various concentrations of andrographolide (0, 12.5, 25, and 50 µM), which were then incubated for 24 h at 37°C with 5% CO_2_. After incubation, medium with 0.1% FBS was replaced, and the cell monolayer was scratched to create a wound gap using a sterile 200-µl micropipette tip, and the cells were further incubated for 12 h. Images were captured by a phase contrast inverted microscope (Olympus model IX83, Shinjuku, Tokyo, Japan) at 0 and 12 h. Cell migration index was obtained from measurement of the wound length by Image J software (version 1.47V, NIH image, National Institutes of Health, Bethesda, MD, USA), relative to the untreated control condition.

### Matrigel Invasion Assay

CCA cells were cultured in 6-well plates at a density of 5 × 10^5^ cells in 4 ml of medium and then maintained at 37°C with 5% CO_2_ for 48 h before being treated with 0, 12.5, 25, and 50 µM of andrographolide for 24 h. Following the incubation, both treated and untreated cells were washed with ice-cold PBS and harvested. The pre-treated and control cells were suspended in serum-free medium at a concentration of 1 × 10^5^ cells in 200 µl and were added to the insert of Matrigel-coated PET membrane (24-well insert, 8-µM pore size; Millipore, Bradford, MA). The inserts were placed into the 24-well plate containing medium supplemented with 10% FBS and incubated at 37°C with 5% CO_2_ for 24 h. Cells that did not invade through the pore were removed by scraping with sterile cotton swab. The invading cells were fixed in 30% methanol for 20 min and stained with 0.1% crystal violet for 20 min prior to counting under a light microscope. Cell invasion index was determined relative to the cells in the control condition.

### Western Blot Analysis

Cells were grownon 10-cm Petri dish at 2 × 10^6^ cells in 10 ml of medium and then cultured for 48 h in 37°C with 5% CO_2_. After incubation, cells were treated with different concentrations of andrographolide for 24 h. Following the treatment, they were washed with ice-cold PBS and collected. Harvested cells were lysed with ice-cold lysis buffer containing 150 mM of NaCl, 1% Triton X-100, 0.5% sodium deoxycholate, 0.1% SDS, and 50 mM of Tris, pH 8.0. Cell lysate was centrifuged at 12,000 × g for 5 min at 4°C to yield whole-cell extract in supernatants. The protein concentration was quantitated using the Bradford assay. Protein samples of 50 µg per lane were loaded onto 10% SDS–PAGE and transferred onto nitrocellulose membranes (Bio-Rad, Hercules, CA, USA). The membranes were blocked for 30 min at room temperature using skim milk powder (Merck Millipore, Billerica, MA, USA) (2% w/v). After being blocked, the membranes were probed with the primary antibodies against protein of interest overnight at 4°C at a dilution recommended by the manufacturers. After being washed, membranes were then incubated with horseradish peroxidase (HRP)-conjugated anti-rabbit or anti-mouse secondary antibodies at a dilution of 1:500 for 1.5 h at room temperature. The immunoreactivities were detected by 3,3′,5,5′-tetramethylbenzidine (TMB) as a substrate of HRP. The intensity of protein bands was evaluated using Image J software. All experiments were performed in three independent experiments. The intensities of protein bands were normalized with the internal control.

### Immunofluorescence

KKU-M213 cells were cultured at 1.5 × 10^5^ cells/ml in 24-well plates and treated with 50 µM of andrographolide for 24 h. The treatment with 1% DMSO served as a control. After incubation, cells were fixed with 4% paraformaldehyde for 20 min at room temperature and permeabilized with 0.1% Triton X-100 for 20 min. After being washed, cells were blocked with PBS containing 2% bovine serum albumin for 1 h and were then incubated with claudin-1 or Snail antibody (1:100) at 4°C overnight. After being washed, the KKU-M213 cells were subjected to the secondary antibody conjugated with Alexa Fluor 594 (1:200) for 1 h at room temperature; then 5 µg/ml of Hoechst 33342 was used to stain nucleus for 20 min. Images were obtained from an inverted microscope.

### Real-Time Reverse Transcription (RT)-PCR

Total RNA was extracted by RNeasy Mini Kit (Qiagen, Hilden, Germany) according to the manufacturer’s protocol. Two micrograms of each RNA sample was reverse transcribed using RevertAid Reverse Transcription (Thermo Fisher Scientific, Waltham, MA, USA). Synthesized cDNA was used in MX 3000p qPCR System (Agilent Technologies, Santa Clara, CA, USA) experiment using SYBR Green Master Mix (PCR Biosystems, London, UK) and analyzed with Software according to the manufacturer’s instruction. The real-time PCR conditions were 40 cycles of 95°C for 2 min, followed by 59°C for 30 s and 72°C for 30 s. Actin gene was used as an internal standard to normalize the amount of total RNA expression in each reaction.

### Treatment With p-38 and JNK-Specific Inhibitors

To verify the role of p-38 and JNK pathways in cell migration that mediates through Snail and claudin-1, the antagonists of p-38 and JNK were used to perform the experiment. KKU-M213 cells were pre-treated with 50 µM of andrographolide or DMSO (control) for 24 h before being incubated with p-38 MAPK inhibitor SB 203580 (5 µM) and JNK inhibitor SP600125 (10 µM) for 30 min. The control cells were pre-treated with 1% DMSO. Then, the cells were replaced with serum-free Ham’s F-12 and further incubated for 3 h. After incubation, they were subjected to wound healing assay and Western immunoblotting.

### Small Interfering RNA Targeting of Claudin-1

Synthetic small interfering RNAs (siRNA) were purchased from Santa Cruz Biotechnology (Santa Cruz, CA, USA). The target DNA sequence of *claudin-1* was GAGGATTACTCCTATGCCGG, and siRNA for *claudin-1* was 5′-GGAUUUACUCCUAUGCCGGtt-3′ and 5′-CCGGCAUAGGAGUAAAUCCtc-3′. KKU-M213 cell lines were transfected with siRNA using RNAimax Lipofectamine Reagent (Invitrogen) according to manufacturer’s instruction. For each experiment, 9 µl of Lipofectamine was mixed with 300 µl of Opti-MEM medium and 4 µl of 10 pmol/µl *claudin-1* siRNA, and then the mixture was pre-incubated at room temperature for 20 min. The cells were washed twice with 2 ml of serum-free Ham’s F-12 and incubated with the pre-incubated mixture containing 1.7 ml of serum-free Ham’s F-12 for 24 h at 37°C with 5% CO_2_. After incubation, the medium with siRNA was removed and replaced with 10% FBS Ham’s F-12 medium, and then the cells were further incubated for 24 h. Then migration assay and detection of *claudin-1* expression using real-time RT-PCR and Western blot analysis were performed.

### Statistical Analysis

Data were representatives of at least three independent experiments. Statistical analysis was performed using two-tailed unpaired Student’s *t*-test and one-way ANOVA and expressed as mean ± standard error of mean (SE) of triplicate measurements. The GraphPad Prism version 6.0 software was used for statistical analysis. Significant value cutoffs were set at *p* < 0.05.

## Results

### Andrographolide Attenuates Migration and Invasion of CCA Cells

**Figure 1:**
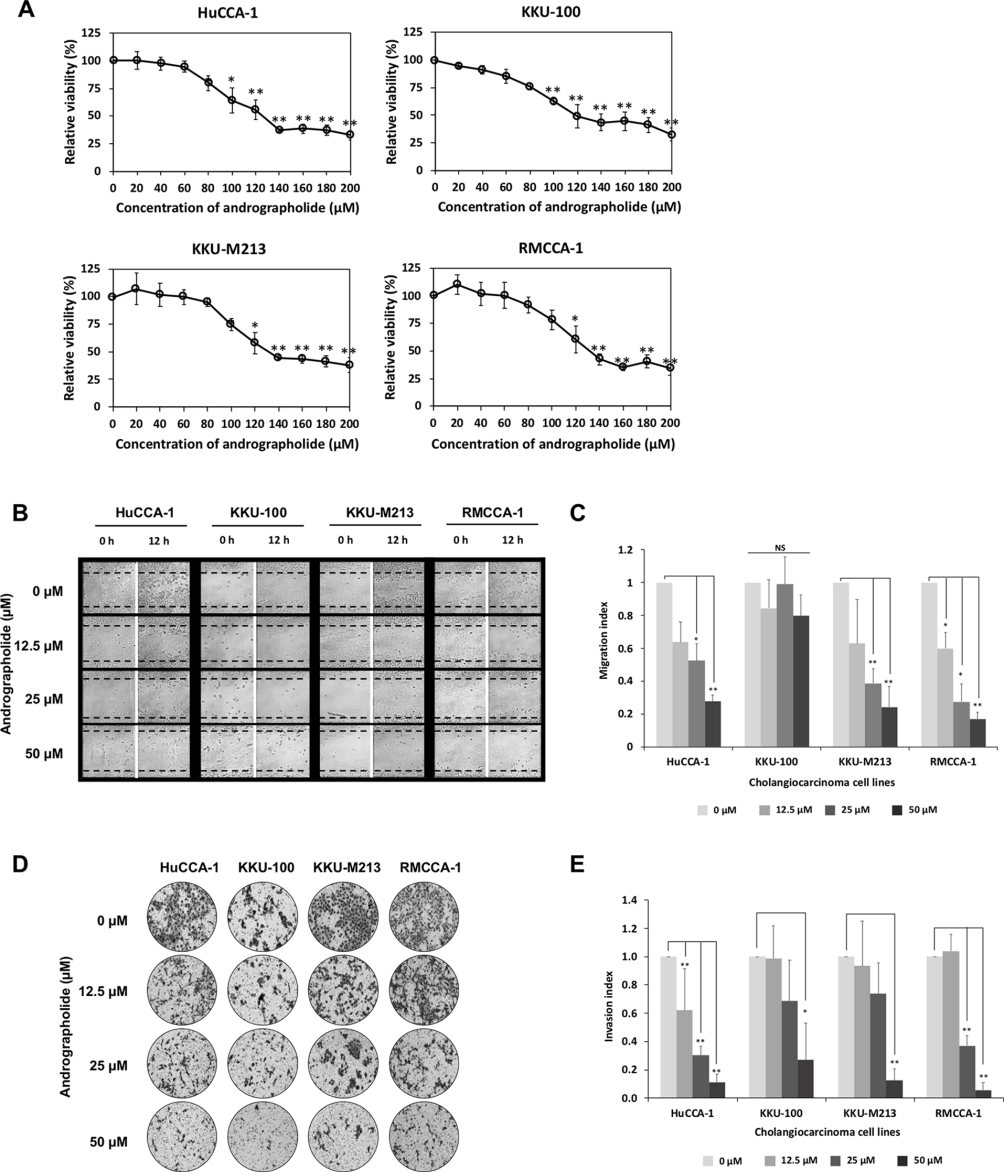
Andrographolide inhibits cholangiocarcinoma (CCA) cell migration and invasion. **(A)** Effect of andrographolide on CCA cell viability. CCA cells were treated with various concentrations of andrographolide (0–200 µM) for 48 h, and cell viability was determined by 3-(4,5-Dimethylthiazol-2-yl)-2,5-diphenyltetrazolium bromide (MTT) assay. The results represented the average of percentage cell viability compared with that of the control. **(B)** CCA cell monolayer treated with andrographolide at the concentrations of 0, 12.5, 25, and 50 µM was scratched, and the size of the wound was photographed at 0 and 12 h following the wound scratch. **(C)** Migration index as indicated by quantitative assessment of wound length. The results represented the average value of migration index compared with that of the control. **(D)** Invasive ability of CCA cells with andrographolide treatment (0, 12.5, 25, and 50 µM) was observed using Matrigel invasion assay. After 24-h incubation, the invasive cells were photographed. **(E)** The number of invasive cells were counted and calculated to invasion index. The results showed the average invasion index compared with that of the control. Data were presented as mean ± SE, which were derived from three independent experiments, *p < 0.05, **p < 0.01.

First, we determined the effect of andrographolide on cell viability in four CCA cell lines including HuCCA-1, KKU-100, KKU-M213, and RMCCA-1. The results indicated that andrographolide significantly reduced CCA cell viability in a concentration-dependent manner. The 50% inhibitory growth concentration (IC_50_) values of andrographolide for CCA cells were 121, 137, 134, and 134 µM for HuCCA-1, KKU-100, KKU-M213, and RMCCA-1, respectively (**Figure 1A**). Our results indicate that andrographolide reduced the CCA cell viability and could induce cell apoptosis. With negligible cytotoxic effect, andrographolide concentration at maximum of 50 µM was used in all subsequent experiments.

The anti-migratory effect of andrographolide was investigated in CCA cells by wound healing assay. Cells were treated with sub-toxic doses of andrographolide (0, 12.5, 25, and 50 µM) for 24 h. **Figure 1B** shows representative photographs of the CCA cell migration. Quantitative analysis revealed that migration indices substantially decreased at 25 and 50 µM in HuCCA-1, KKU-M213, and RMCCA-1, but not in KKU-100 cells (**Figure 1C**), suggesting that andrographolide inhibited CCA cell migration in a dose-dependent manner. Consistent with previous observations, KKU-100 did not seem to exhibit migration activity. Furthermore, CCA cell invasion was determined using a Matrigel-coated transwell. **Figure 1D** illustrates the effect of andrographolide on CCA cell invasion. The quantitative data also showed that andrographolide inhibited the ability of CCA cell invasion in a dose-dependent manner (**Figure 1E**). Our data demonstrated that andrographolide suppressed the ability of migration and invasion in CAA cells.

### Andrographolide Inhibits Claudin-1 Expression in CCA Cells

**Figure 2:**
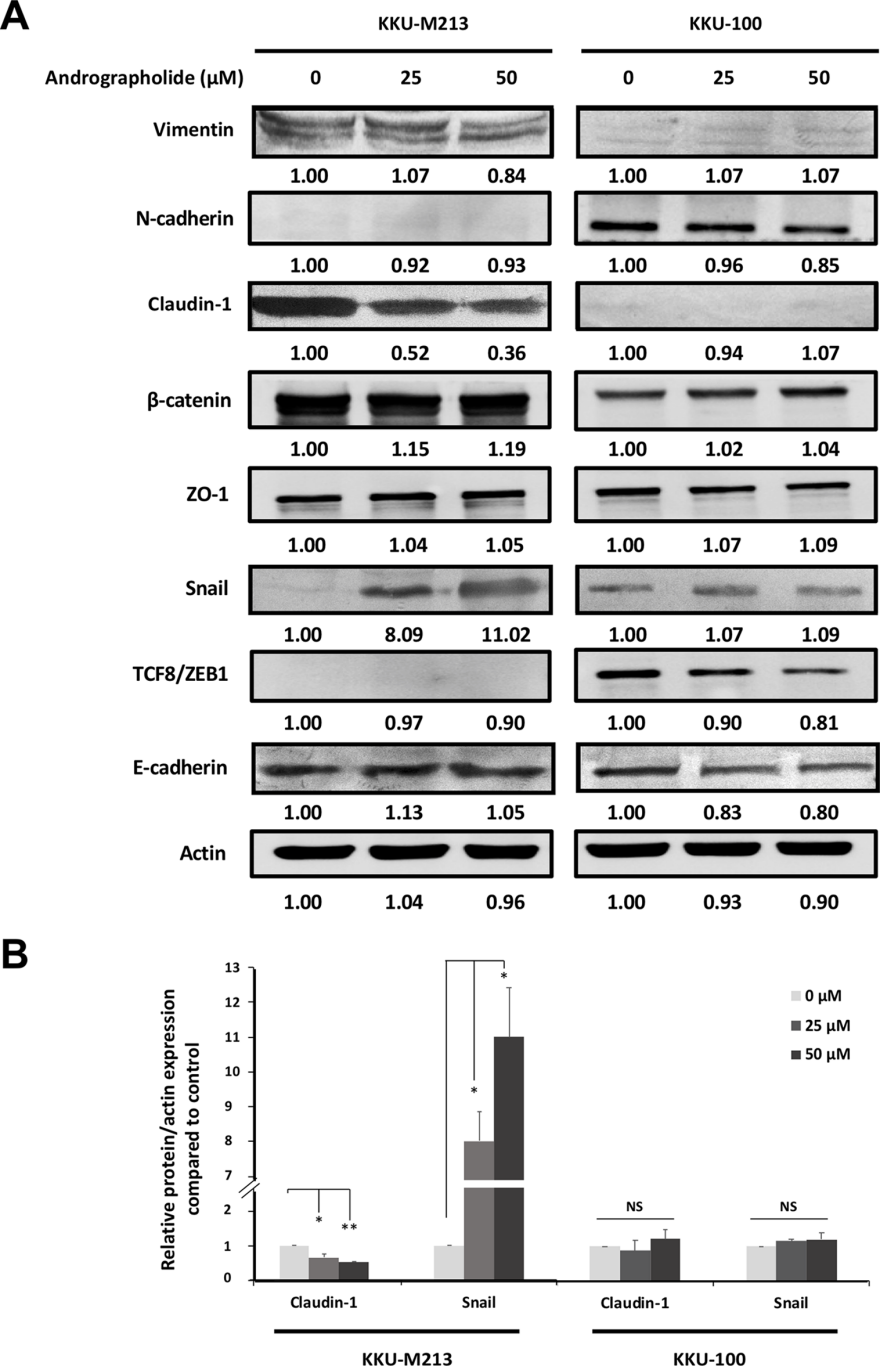
Effects of andrographolide on the expression of epithelial–mesenchymal transition (EMT) proteins in CCA cells. **(A)** KKU-M213 and KKU-100 cells were treated with 0, 25, and 50 µM of andrographolide for 24 h prior to harvesting proteins. The expression of EMT-related proteins was evaluated using Western immunoblotting with actin as internal control. Representative results of three independent experiments are shown. The level of protein expression was quantified by intensitrometric analysis relative to actin as ratios to the untreated control. **(B)** The graphs present the expression of claudin-1 and Snail in KKU-M213 and KKU-100 upon the treatment of andrographolide. The results of bar graph are represented by the average of relative intensity compared with that of the control. Data were presented as mean ± SE, which were derived from three independent experiments, *p < 0.05, **p < 0.01.

**Figure 3:**
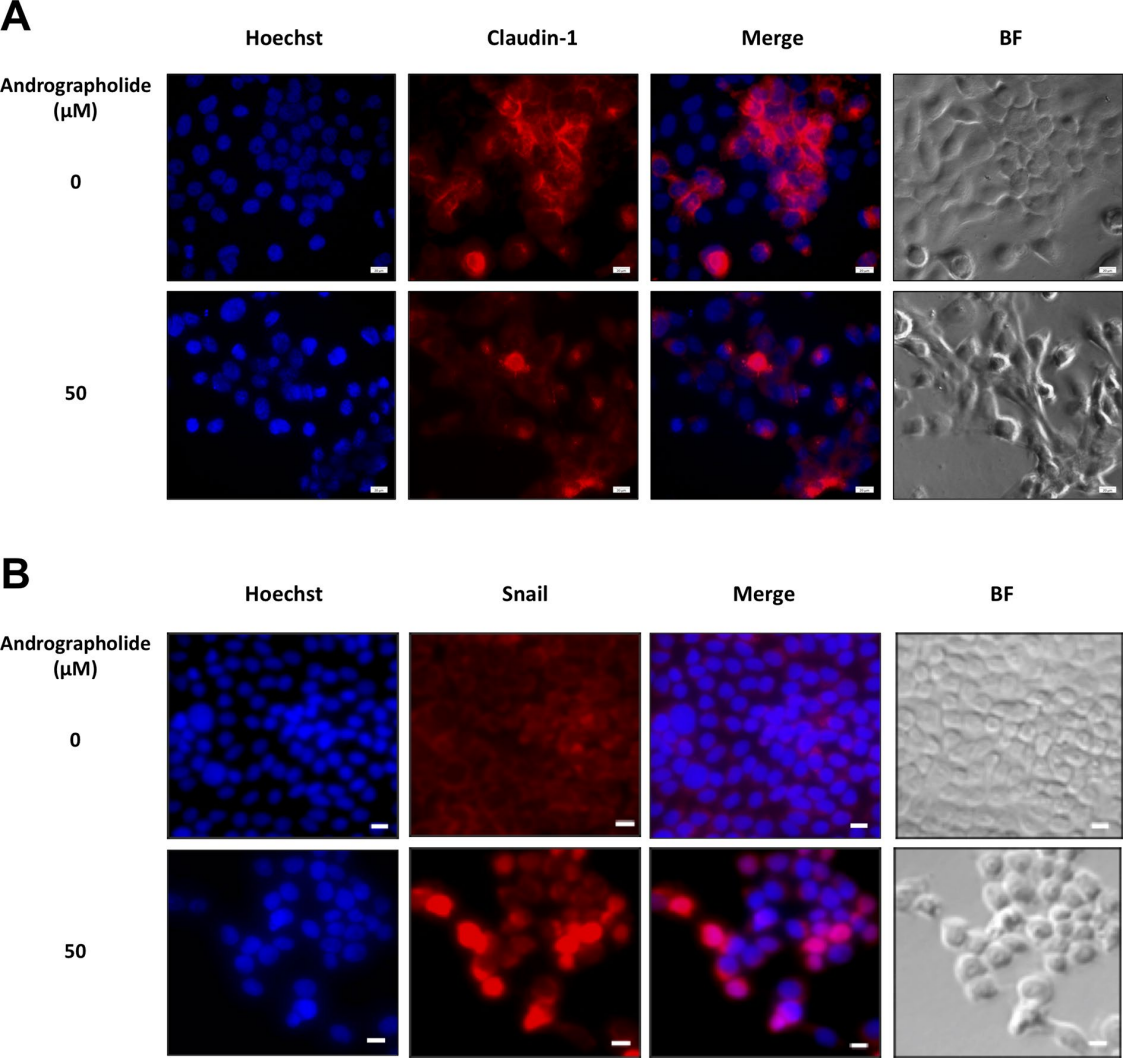
Immunofluorescence of claudin-1 and Snail in KKU-M213 cells following andrographolide treatment. KKU-M213 cells were treated with 50 µM of andrographolide prior to fixing and staining with **(A)** claudin-1 and **(B)** snail antibodies. The control cells were treated with 1% DMSO. Anti-mouse secondary antibody conjugated with Alexa Fluor 594 was added (red). Hoechst 33342 was used as nuclei counterstain (blue). The images were obtained from Olympus IX83 inverted microscope.

To investigate the mechanism through which andrographolide inhibits CCA cell migration and invasion, we analyzed the expression of EMT proteins including vimentin, E-cadherin, claudin-1, and Snail. In this experiment, we used two cell lines, migratory KKU-M213 and non-migratory KKU-100 cells. The results showed that claudin-1 expression was substantially decreased in KKU-M213 cells upon exposure to andrographolide treatment, whereas the expression of Snail, a repressor of claudin-1, was up-regulated than was control. No apparent changes of claudin-1 and Snail were observed in KKU-100 cells (**Figure 2**). There were no significant changes in the expression of other EMT-related proteins following the exposure to andrographolide in both cell types. The effect of andrographolide on claudin-1 and Snail expression in KKU-M213 cells was confirmed by immunofluorescence (**Figure 3**). From these data, andrographolide altered the expression of claudin-1 and Snail in migratory KKU-M213 cells but not in non-migratory KKU-100 cells, suggesting that andrographolide attenuated motility of CCA cells through claudin-1 and Snail expression.

### Suppression of Claudin-1 Expression Inhibits Cell Migration of CCA Cells

**Figure 4:**
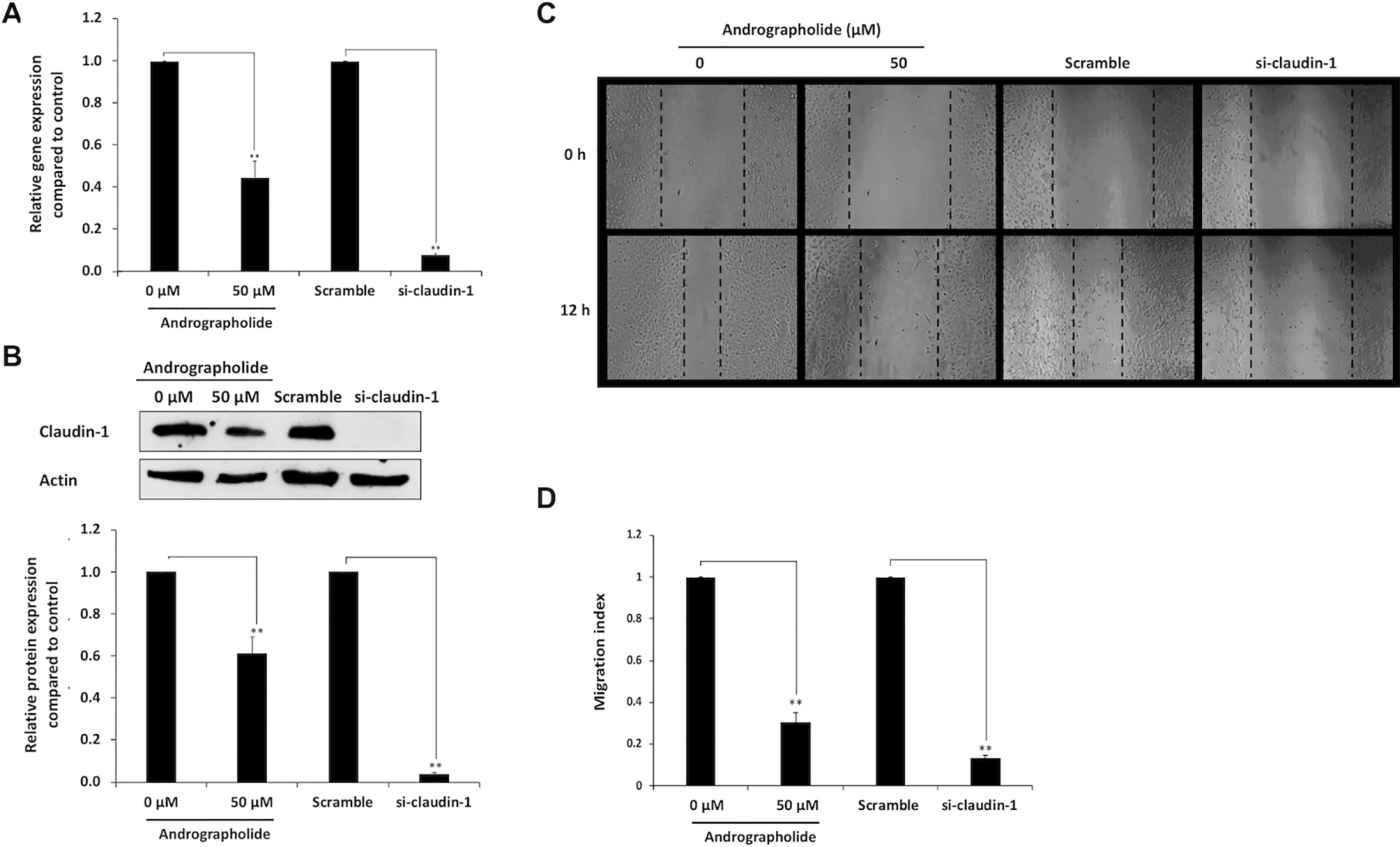
Inhibitory effect of andrographolide on cell migration mediated by claudin-1. The RNA interference of claudin-1 was used to knock down the claudin-1 gene in KKU-M213 cells. KKU-M213 were treated with 0 µM (1% DMSO), 50 µM of andrographolide, scramble, and si-claudin-1 prior to harvesting RNA and cellular proteins for the detection of claudin-1. **(A)** The expression of claudin-1 mRNA from real-time RT-PCR; **(B)** The level of claudin-1 protein from Western blot analysis. Actin was used as internal standard. **(C)** With those treatments, KKU-M213 was scratched to make a wound and photographed at 0 and 12 h to measure the wound length for migration index analysis. **(D)** The results of bar graph are represented by the average of relative intensity compared with that of control. Data were derived from three independent experiments and are presented as mean ± SE, *p < 0.05, **p < 0.01.

Transient suppression of claudin-1 in KKU-M213 cells was performed using siRNA specific to claudin-1. Cells treated with scramble RNA were used as controls. The real-time RT-PCR and Western blot analysis showed that claudin-1, compared with the controls, was successfully knocked down by its specific siRNA >8-folds (**Figures 4A, B**). Migration activity of KKU-M213 cells was evaluated by wound healing assay. The results revealed that the cells treated with si-claudin-1 exhibited significant reduction in migration activity than did the control cells treated with scramble RNA. Similar observations were found in the condition of andrographolide treatment, which were consistent with the previous experiments (**Figures 4C, D**). The data demonstrated that claudin-1 plays an important role in CCA cell migration.

### Role of the p-38 MAPK Pathway in the Andrographolide-Mediated Inhibition of Cell Migration and Claudin-1 Expression

**Figure 5:**
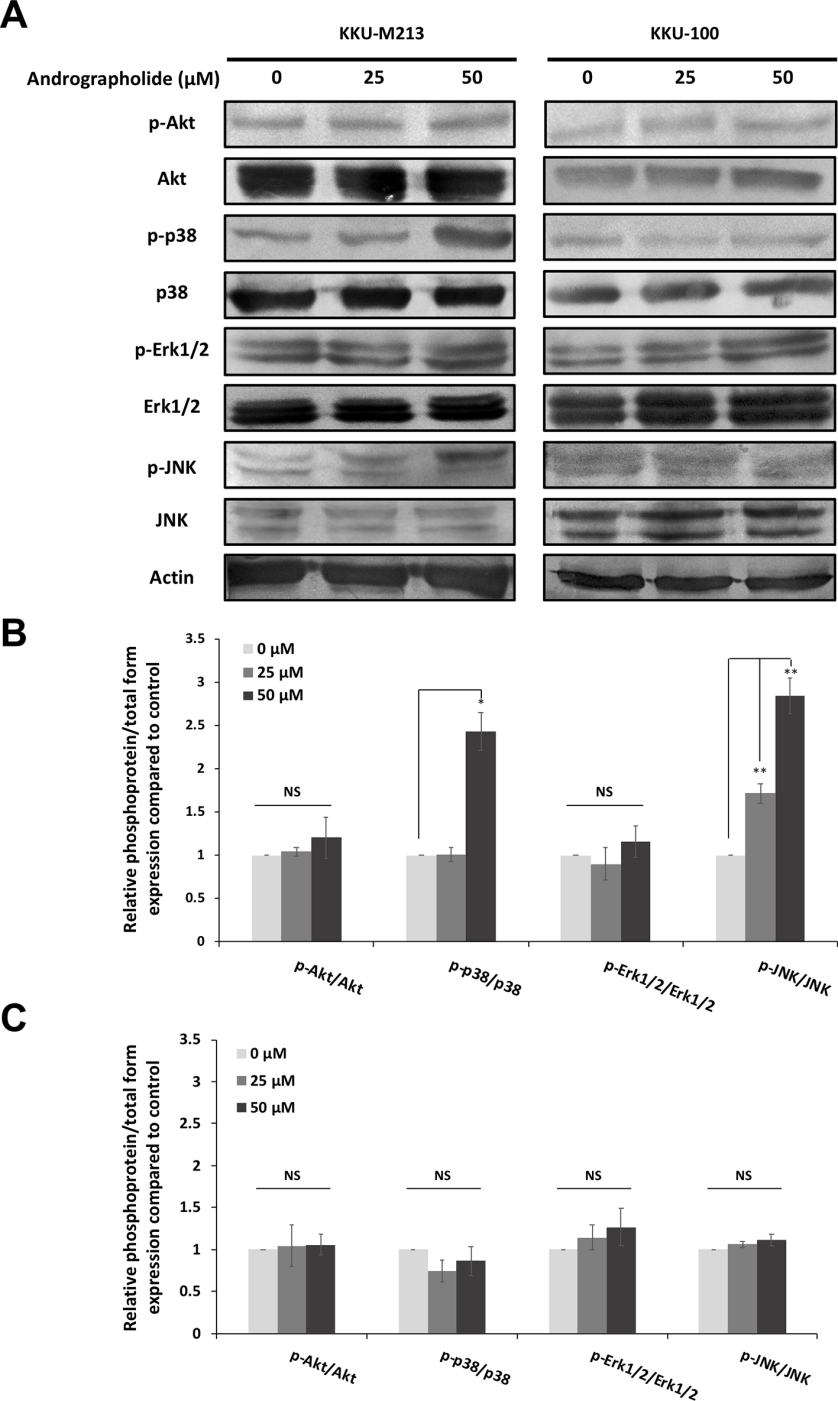
Andrographolide induced phosphorylation of p-38 and JNK in KKU-M213 cells. **(A)** KKU-M213 and KKU-100 cells were treated with 0, 25, and 50 µM of andrographolide for 24 h. Cellular proteins were analyzed for the expression of phospho-Akt (p-Akt) (Ser473), Akt, and MAPK signaling pathways including phospho-p-38 (p-p-38) (Thr180/Tyr182), p-38, phospho-Erk1/2 (p-Erk1/2) (Thr202/Tyr204), Erk1/2, phospho-JNK (p-JNK) (Thr183/Tyr185), and JNK. Actin served as an internal control. The levels of protein expression in **(B)** KKU-M213 and **(C)** KKU-100 cells were quantified by intensitrometric analysis relative to the control. The results of the bar graph are represented by the average of relative intensity compared with that of the control. Data were derived from three independent experiments and are presented as mean ± SE, *p < 0.05, **p < 0.01.

To elaborate further on the potential mechanism by which andrographolide inhibits CCA cell migration, the signaling pathways including MAPK and Akt pathways were determined under andrographolide treatment. The results showed that andrographolide enhanced phosphorylation of p-38 MAPK and JNK in KKU-M213 cells, but no alteration was observed in KKU-100 cells. However, andrographolide did not affect the expression of Akt and Erk1/2 in both KKU-M213 and KKU-100 cells (**Figure 5**). At 50 µM of andrographolide, significant induction of phosphorylation of p-38 MAPK and JNK was observed, suggesting that andrographolide increased the activation of p-38 MAPK and JNK. It is possible that they further regulate the downstream target proteins involving in migratory inhibition of CCA.

**Figure 6:**
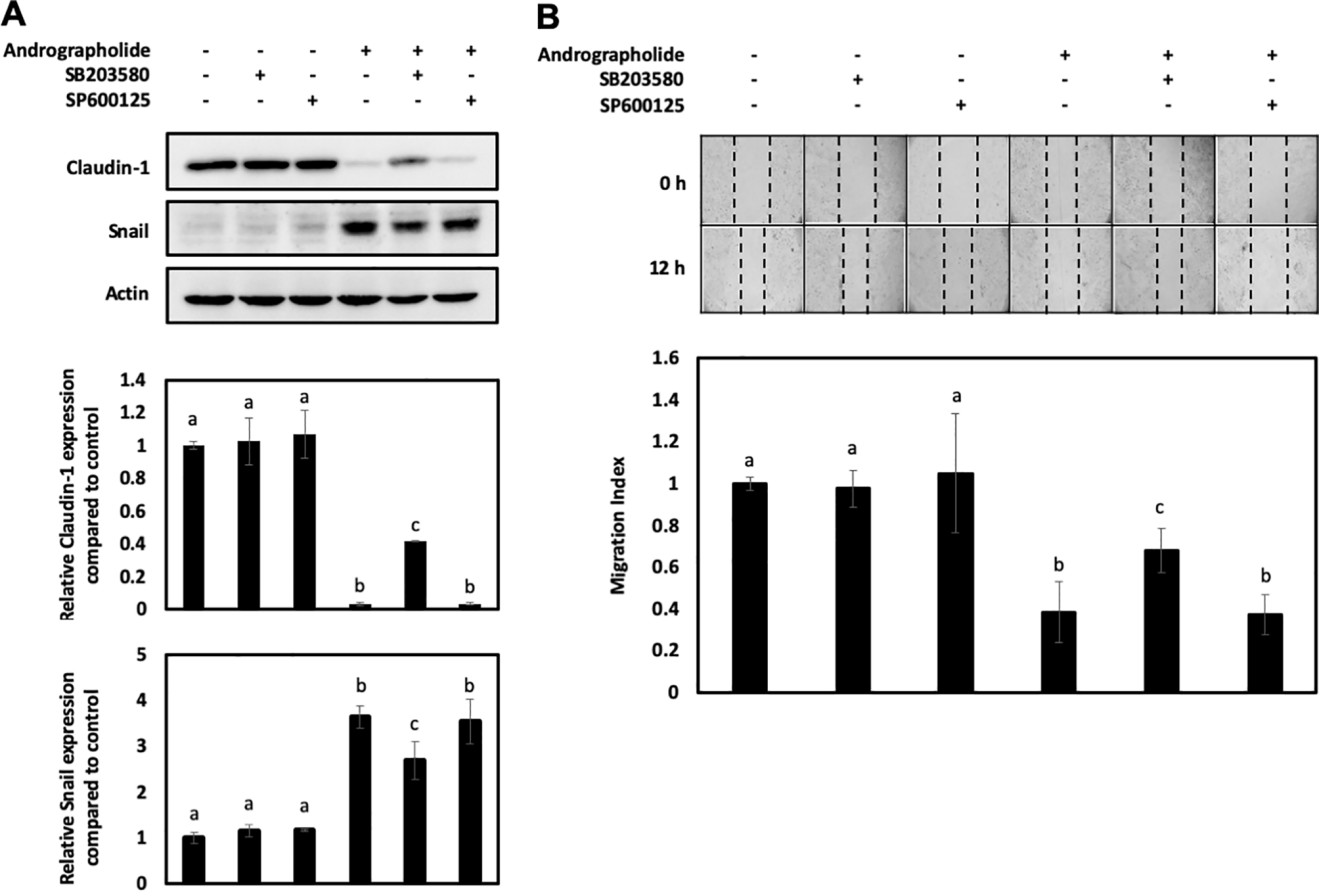
Effect of p-38 MAPK inhibitor (SB 203580) and JNK inhibitor (SP500125) on KKU-M213 treated with andrographolide. **(A)** KKU-M213 was treated with 50 µM of andrographolide or DMSO control for 24 h prior to the treatment with the inhibitors. Proteins from cell lysate were analyzed for the expression of claudin-1 and Snail, and actin was used as an internal control. **(B)** KKU-M213 cells with treatment of andrographolide and the inhibitors were scratched and photographed at 0 and 12 h to measure the wound length for migration index analysis. The results of bar graph are represented by the average of relative intensity compared with that of the control. Data are presented as mean ± SE, which were derived from three independent experiments. Lowercase letters similar between treatments represent no statistically significant difference.

To verify the relationship among the andrographolide-mediated inhibition of claudin-1 and p-38 MAPK and JNK activation, the KKU-M213 cells were treated with the specific inhibitors of p-38 MAPK (SB 203580) and JNK (SP600125). The results revealed that andrographolide treatment followed by p-38 MAPK inhibitor could recover the expression of claudin-1 but suppressed Snail expression than did the cells exposed to andrographolide only (**Figure 6A**). Moreover, p-38 MAPK inhibitor reversed the effect of andrographolide on CCA cell migration (**Figure 6B**). However, this notion could not be observed with the JNK inhibitor. Taken together, these results indicate that claudin-1 and Snail might be key proteins downstream of p-38 MAPK, which play a role in regulating the migration of CCA cells.

## Discussion

Because of high mortality rate and lack of effective curative options in CCA, the need for novel therapeutic measures are urgently needed. In recent years, a number of studies have indicated that the naturally extracted compounds from herbal plants exhibit anticancer properties. Andrographolide, a major constituent from *A. paniculata*, has been shown to have multi-functions of anticancer activities in variety of cancer types (Lim et al., 2012; Mishra et al., 2015). The crude extract of *A. paniculata* displays the ability to arrest cell cycle and induce apoptosis in CCA cells (Suriyo et al., 2014). However, the effects of purified andrographolide on other relevant CCA properties including cell migration and invasion have never been described. This current study hence explored such effect together with underlying molecular mechanism using four different CCA cell lines.

Andrographolide has been shown to inhibit growth in several cancer cell types such as breast cancer, colon cancer, lung cancer, melanoma, leukemia, prostate cancer, and ovarian cancer (Rajagopal et al., 2003). We first provided evidence that andrographolide significantly suppressed all CCA cell proliferation. The pro-apoptotic effect of andrographolide has been associated with various mechanisms depending on the cell types. While andrographolide enhanced the expression of Bax, caspase-3, and caspase-9, it down-regulated that of Bcl-2, leading to apoptosis in melanoma B16F-10 cells (Pratheeshkumar et al., 2012), and it promoted apoptosis in pancreatic cancer cells *via* inhibition of STAT3 and Akt activation (Bao et al., 2013). In addition, the analogue form of andrographolide has previously been shown to induce apoptosis by mediating topoisomerase II alpha in KKU-M213 cells (Nateewattana et al., 2014).

We showed that andrographolide effectively inhibited the ability of cell migration and invasion in CCA cell lines at sub-cytotoxic doses, except for KKU-100 cells, which did not migrate and had a low capacity of invasion than had other cell lines in our experimental settings. KKU-100 was established from poorly differentiated tubular adenocarcinoma with low expression of CEA and CA-125 compared with the primary tumor (Sripa et al., 2005). KKU-100 cells exhibited high expression of thymosin 10 (Tβ10), a protein that inhibits actin polymerization, which is commonly found in primary tumors but decreases in metastatic tumor cells, causing a low capacity in migration (Sribenja et al., 2013). The molecular mechanism underlying the andrographolide-mediated inhibition of CCA cell migration and invasion was then characterized. We found that andrographolide suppressed the level of claudin-1 protein in high migratory CCA cells. Claudin-1 is a protein involved in the EMT pathway that functions as a major tight junction protein. It functions by both promoting and suppressing tumors depending on cancer types (Kwon, 2013). However, several migratory and invasive cancerous cell types have been associated with high expression of claudin-1 including breast cancer, colon cancer, liver cancer, oral squamous carcinoma, and melanoma (Leotlela et al., 2007; Akasaka et al., 2010; Yoon et al., 2010; Blanchard et al., 2013). In contrast, andrographolide promoted the expression of Snail in a dose-dependent manner. Snail is an EMT transcriptional repressor that has been reported as a negative regulator of epithelial proteins including claudin-1 (Martínez-Estrada et al., 2006). Interestingly, the expression of claudin-1 and Snail was altered only in migratory KKU-M213 cells, but not in less motile KKU-100 cells, suggesting that andrographolide affects migration-mediated proteins. Furthermore, the knockdown of claudin-1 in KKU-M213 cells caused the reduction in cell migration than did control. These findings were similar to the effect of andrographolide on the reduction of claudin-1 expression and the inhibition of cell migration in migratory CCA cells.

The intracellular signaling pathways including Akt and MAPK have been implicated in cancer cell migration and invasion (Jin et al., 2017). Our results revealed that andrographolide caused a dose-dependent increase in the cellular level of phosphorylated p-38 MAPK and JNK in KKU-M213, but not KKU-100. However, there was no noticeable change in total and phosphorylated levels of Akt and Erk1/2 at the same dosages. To further elucidate the related effects of andrographolide on KKU-M213 cells, we explored the effect of andrographolide combined with specific inhibitors of the p-38 MAPK and JNK on cell migration. We observed that the combined treatment of andrographolide and the p-38 MAPK inhibitor recovered the expression of claudin-1 and migration ability, but this effect was not observed in the treatment with the JNK inhibitor. Nevertheless, the activation of JNK upon the treatment with andrographolide might play a role in other cellular mechanisms.

**Figure 7:**
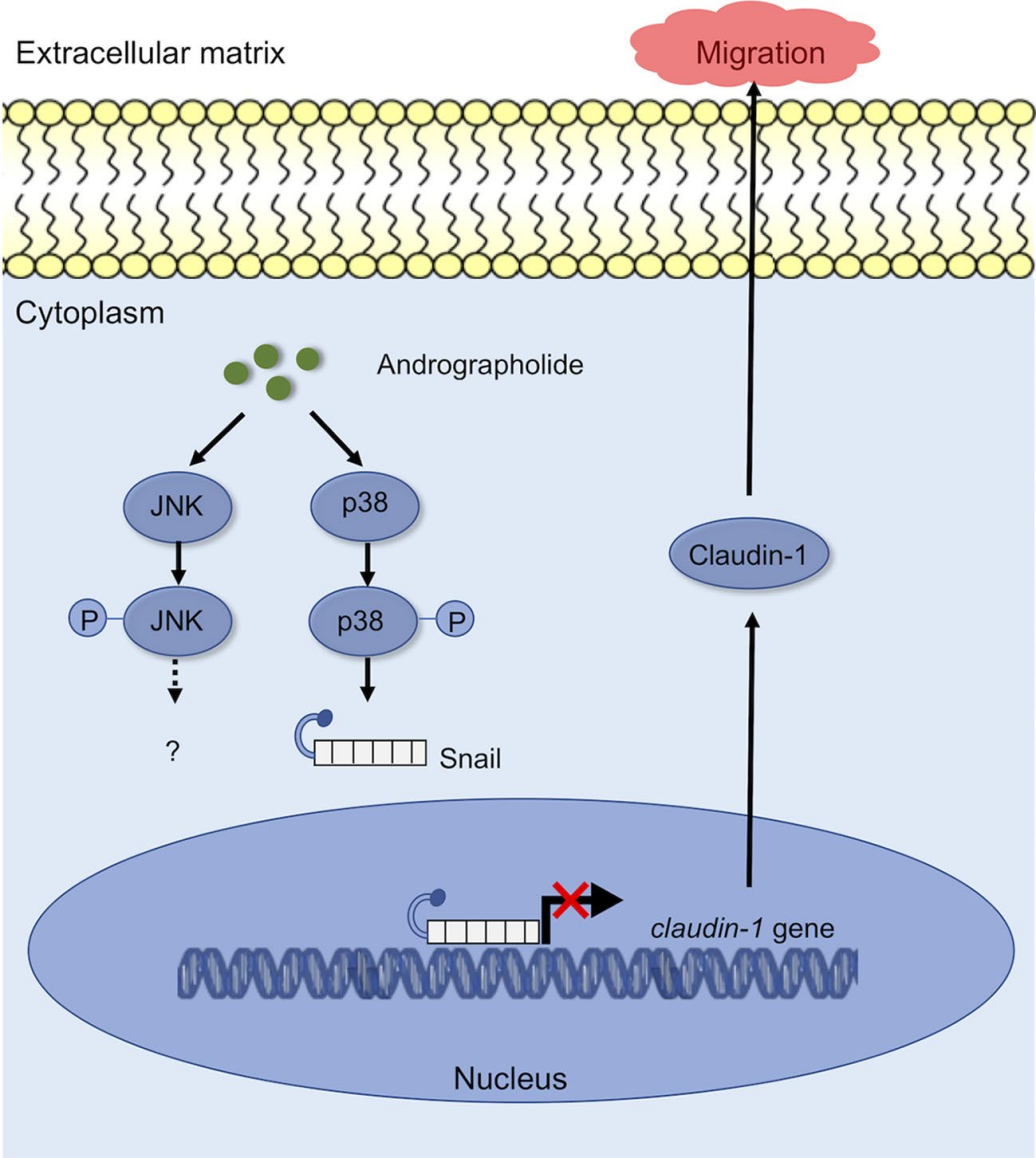
A working model showing the ability of andrographolide to regulate the migration ability of CCA cells.

In the present study, we proposed a schematic presentation of possible mechanisms for the inhibitory effect of andrographolide on migration of CCA cells (**Figure 7**). Andrographolide inhibits CCA cell migration through the activation of p-38 MAPK, leading to promotion of Snail and repression of claudin-1. Further investigation including animal studies and the rescue experiments are warranted to provide more support to our current findings. Taken together, the findings from this study highlighted the potential inhibitory effects of andrographolide on CCA cells and also revealed its precise molecular mechanism of anti-migration property. Andrographolide therefore serves as a good promising agent for CCA therapeutic development.

## Data availability

The raw data supporting the conclusions of this manuscript will be made available by the authors, without undue reservation, to any qualified researcher.

## Author Contributions

PP performed experiments, analyzed the data, and wrote the manuscript; SK and SO contributed to the experimental work and analyzed the data; TJ conceived and designed the study, analyzed the data, wrote the manuscript, and supervised the research. All authors read and approved the final version of the manuscript.

## Funding

This work is supported by grants from Cerebos foundation, Medical Research Council (MRC) through UK-Thailand Research Collaborations (Newton Fund), the Thailand Research Fund (grant no. DBG5980006) and Faculty of Science, Mahidol University (TJ). PP is funded by the Science Achievement Scholarship of Thailand. The equipment was facilitated by Central Instrument Facility (CIF) at Faculty of Science, Mahidol University.

## Conflict of Interest Statement

The authors declare that the research was conducted in the absence of any commercial or financial relationships that could be construed as a potential conflict of interest.
